# Benchmark data for sulcal pits extraction algorithms

**DOI:** 10.1016/j.dib.2015.10.004

**Published:** 2015-10-21

**Authors:** G. Auzias, L. Brun, C. Deruelle, O. Coulon

**Affiliations:** a Institut de Neurosciences de la Timone, UMR7289, Aix-Marseille Université & CNRS, Marseille, France; b Laboratoire des Sciences de l׳Information et des Systèmes, UMR7296, Aix-Marseille Université & CNRS, Marseille, France

## Abstract

This article contains data related to the research article Auzias et al. (2015) [1]. This data can be used as a benchmark for quantitative evaluation of sulcal pits extraction algorithm. In particular, it allows a quantitative comparison with our method, and the assessment of the consistency of the sulcal pits extraction across two well-matched populations.

Specifications Table

**Table:** 

Subject area	*Biology*
More specific subject area	*Neuroimaging, cortical surface, morphometry*
Type of data	*Table, reconstructed cortical surface meshes, sulcal depth textures, sulcal pits resulting from (Auzias et al., 2015)*[1]
How data was acquired	*n/a*
Data format	*Meshes and textures are in gifti file format (.gii), Subjects ID and phenotype are given in a table*
Experimental factors	*Anatomical MRI from selected subset of the public database OASIS were processed using freesurfer. Cortical surfaces quality was controlled. Sulcal pits and depth textures were computed as described in Auzias et al., 2015*[1]
Experimental features	*n/a*
Data source location	*Marseilles, France*
Data accessibility	*Data is provided in Data in Brief DataVerse:*https://dataverse.harvard.edu/dataset.xhtml?persistentId=doi:10.7910/DVN/PRK2U1

Value of the data

• **The sulcal pits, defined as the deepest points lying in each fold of the cortical surface, are gathering growing interest in the literature** [1], [2], [3], [4], [5], [6], [7], [8].
• **The data presented here consist of well characterized subjects with high-quality cortical meshes, divided in two subgroups carefully matched according to several parameters, with the depth map and sulcal pits resulting from the extraction procedure proposed in** [1].
• **These data can be used for the benchmarking of new sulcal pit extraction algorithms, with a quantitative comparison with** [1]**, and for the assessment of the consistency of the sulcal pits across two well matched populations.**

## 1 Data

This data article contains data related to the research article [1] and can serve as benchmark dataset for quantitative evaluation of sulcal pit extraction algorithm. Defined as the deepest points lying in each fold of the cortical surface, the recently introduced geometric landmarks called “sulcal pits” are gathering growing interest as shown by the increasing number of related publications [1], [2], [3], [4], [5], [6], [7], [8]. If their extraction if fairly simple on principle, algorithms are relying on a few parameters that are essential for the filtering of spurious pits. Such parameters are difficult to tune, and their value can have a large influence on the performance of algorithms. In [1], we proposed a method for the extraction of sulcal pits from a reconstructed cortical surface tessellation, that included a framework for the optimization of such parameters. The validation of this technique was performed on two different groups of healthy subjects with carefully matched characteristics, in order to assess the reproducibility of the technique across groups. We here make available to the community all the data needed to run benchmark analyses. The data consists of 1) high quality cortical surface meshes, 2) the corresponding spherical representation of each cortical surface needed to compute group-level statistics, 3) the corresponding cortical depth maps (DPF), 4) the sulcal pit maps, 5) the population phenotype and identification number in the original database (OASIS).

## 2 Experimental design, materials and methods

A particular attention was drawn to gather a large set of high-quality cortical surface tessellations from a well-controlled population of healthy individuals. For that purpose, 137 right-handed subjects were selected from the Open Access Series of Imaging Studies (OASIS) database (www.oasis-brains.org) [9]. The OASIS cross-sectional dataset has a collection of 416 subjects aged from 18 to 96, including older adults with dementia.

**Fig. 1:**
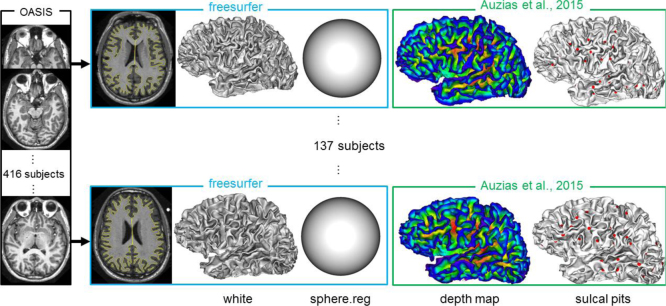
The MRI from a set of 137 subjects were segmented using freesurfer. The depth map and sulcal pits were then computed for each white mesh using the methods presented in [1]. The data provided consist of the white and sphere.reg meshes from freesurfer as well as the depth map and the sulcal pits.

Fig.1 illustrates the processing pipeline used and identifies the data provided with respect to this pipeline. For each subject, three to four individual T1-weighted magnetization-prepared rapid gradient echo (MP-RAGE) scans were obtained on a 1.5 T Vision system (Siemens, Erlangen, Germany) with the following protocol: in-plane resolution=256×256 (1 mm×1 mm), slice thickness=1.25 mm, TR=9.7 ms, TE=4 ms, flip angle=10 u, TI=20 ms, TD=200 ms. Images were motion corrected and averaged to create a single image with a high contrast-to-noise ratio (Marcus et al., 2007) [9]. Anatomical MR images were processed using Freesurfer 5.1.0 (http://surfer.nmr.mgh.harvard.edu), in order to extract the inner cortical surface mesh (white), surface area and intracranial volume, and obtain spherical inter-individual correspondences through the Freesurfer sphere.reg mesh [10], [11]. The accuracy of the cortical surface extraction was visually controlled for each individual. We then defined two groups of 68 and 69 young adult healthy subjects (aged 18–34 yr) matched in age, gender, cortical surface area and intracranial volume (see Table 1). These two groups can be used to evaluate the consistency of the sulcal pits extraction tool across two well controlled populations.

For each cortical mesh, we then computed the Depth Potential Function (DPF) introduced in [12] as an estimation of the cortical fold depth, using a new implementation freely available in BrainVISA 4.5.0 (http://brainvisa.info/). We used this depth map to extract the sulcal pits of each fold using the technique detailed in [1]. Both the DPF and the sulcal pits are provided for each subject in the dataset.

## Figures and Tables

**Table 1:** Phenotype and global morphometric measures that were considered for matching the two groups.

	Group 1 (*N*=68)	Group 2 (*N*=69)	Difference
Age: mean (std)	23.1 (0.42)	23.2 (0.42)	*T*=0.14/*p*=0.88
Gender: M/F	34/34	35/34	*χ*²=0.007/*p*=0.93
Surface area: mean (std)	174,806 (1903) mm²	173,563 (1890) mm²	*T*=−0.46/*p*=0.64
Intracranial volume: mean (std)	1,524,847 (17,136) mm^3^	1,528,069 (17,011) mm^3^	*T*=0.13/*p*=0.89
